# Factors associated with compliance with community directed treatment with ivermectin for onchocerciasis control in Southwestern Ethiopia

**DOI:** 10.1186/1756-3305-3-48

**Published:** 2010-06-02

**Authors:** Daniel Yirga, Kebede Deribe, Kifle Woldemichael, Mekite Wondafrash, Wondosen Kassahun

**Affiliations:** 1 Department of Epidemiology and Biostatistics, Faculty of Public Health, Jimma University, Jimma, Ethiopia; 2 Fayyaa Integrated Development Association-NCMI, PEPFAR-New Partners Initiative, Addis Ababa, Ethiopia; 3 Department of Population and Family Health, Faculty of Public Health, Jimma University, Jimma, Ethiopia

## Abstract

**Background:**

Although ivermectin is distributed free of charge through the African Programme for Onchocerciasis Control (APOC), not all eligible individuals within communities receive the annual treatment. This poses a serious threat to efforts aimed to control onchocerciasis. This study attempts to determine factors associated with compliance to Community Directed Treatment with Ivermectin (CDTI) and provides a basis for trying to understand how best to sustain long-term compliance in order to achieve success in the control of onchocerciasis.

**Methods:**

An unmatched case-control study was conducted in Bebeka coffee plantation southwest Ethiopia. Cases were, compliant i.e., those individuals who had been registered on the relevant treatment registers and had taken all the five annual doses of Ivermectin. Controls were non-compliant, i.e. those individuals who had been recorded in the relevant treatment registers during the first treatment round(2003), and did not take at least two doses of which one being in the last treatment round (2007). Data were collected using a pre-tested interviewer administered structured questionnaire. Data were edited, cleaned, coded and analyzed using SPSS version 12.0.1 for Microsoft Windows. Multiple logistic regression models was used to identify factors associated with compliance to ivermectin.

**Results:**

From the total of 456 individuals selected for administration of the survey questionnaire, 450(225 cases and 225 controls) were contacted and completed the study 2 refused and 4 were unavailable. Five factors associated with compliance were identified: high risk perception [Adjusted Odds Ratio(AOR) = 1.98, 95% Confidence Interval (CI), 1.32-2.95], one's family support [AOR = 1.86, 95% CI, 1.22-2.84], perceiving that the Community Drug Distributors (CDDs) are doing their work well [AOR = 2.84, 95% CI, 1.50-5.37] and perceiving measuring height is the best way to determine a person's treatment dose [AOR = 6.37, 95% CI, 2.10-19.29] are positive predictors of compliance to ivermectin.

**Conclusion:**

Interventions to improve compliance in the area should focus on health education using epidemiological data in order to increase risk perception and dispelling misconceptions. Motivation and continued support to improve CDD's performance including training and incentives are crucial.

## Background

Onchocerciasis, "river blindness", is a parasitic disease caused by a filarial worm, Onchocerca *volvulus*. The disease is transmitted by the blood feeding black fly, of the genus *Simulium *[1]. Onchocerciasis is endemic in many tropical countries but mainly in the equatorial region of Africa. According to recent estimates, 37 million people are infected worldwide and 90 million are at risk in Africa [2].

In Ethiopia, 3 million people are already infected, whereas 7.3 million are at risk of infection and almost everyone in an endemic village will harbor the disease. Nine regions surveyed for river blindness were shown to be endemic; the endemic areas extend from the northwest part to southwest part of the country that borders Sudan [3,4]. The main symptom of the disease in the country is dermal (skin) manifestations that are characterized by disabling intense itching and thickening of the skin, hanging groin etc. Blindness, which is a common manifestation of the disease in West Africa, is a rare complication in Ethiopia, which is located in East Africa [3]. The prevalence of onchocerciasis in the country ranges from 85.3% in Teppi province, southwestern Ethiopia to 6.9% in the Kuwara province of Northwest Ethiopia [5].

Onchocerciasis has been targeted for control, at least as a disease of public health and socio-economic development importance, in Ethiopia along with other 19 African countries [3]. Mass treatment of high risk communities with ivermectin is adopted in line with the African Program for Onchocerciasis Control (APOC)'s Community Directed Treatment with ivermectin (CDTI) strategy, since 1996 [6]. CDTI is in which the community itself has the responsibility for organizing and executing treatment of its members. CDTI is based on the principle of active, structural community participation [7,8]. The process empowers community members to make major decisions and direct the distribution of ivermectin for a sustained period of years. Examples of community decisions made with respect to mass treatment include: dates of distribution; mode of distribution (e.g. house-to-house, central place); persons who will guide distribution; and selection of the community implementers, also known as Community- Directed Distributors (CDDs). In addition to making such planning decisions, communities take responsibility for: conducting a community census, collecting drug supplies, mobilizing members during the drug distribution process, as well as recording treatments provided and coverage attained [9].

The drug ivermectin (trade name, Mectizan^®^) is a microfilaricide and a temporary microfilarial suppressant, which was approved in 1978 for mass treatment of communities exposed to onchocerciasis. Treatment has to be continued annually for at least 10 years in order to cover the life span of the adult worms, which are not killed by this drug [10]. All members of households in the communities will be treated with ivermectin 150 mg/kg body weight in each round provided there are no contraindications. Contraindications for administration of ivermectin include being younger than 5 years or 90 centimeters in height, being pregnant or and lactation of infant less than one week of age, having serious health problem, e.g. asthma, renal or hepatic disease. Before administering the drug, community members will be informed about the disease and possible adverse reactions following therapy as well as the availability of help for any untoward reactions. They are then weighed and asked to swallow the drug on the spot [10,11].

To achieve the target, the treatment must continue for 15-20 years [12] with sustained compliance and community coverage of at least 90% [13]. Together with coverage, compliance was found to be an important factor for the success of the program through assurance of adequate reduction (to control serious onchocerciasis and, eventually, to have a significant impact on transmission) in transmission of the parasite and thus a good degree of protection from further infection [4].

In the African region, several studies showed that mass treatment with ivermectin resulted in a major reduction in transmission of onchocerciasis [14]. However, revival of transmission has been noticed following disruption of ivermectin treatment before an optimal duration of the lifespan of adult O. volvulus worms (ten years above) [1].

The largest trial, undertaken in the hyper-endemic focus of Asubende on the river Pru in Ghana, provided the most detailed information on the effect of mass treatment on transmission, showing a major reduction in vector infectivity but still significant residual transmission after treatment [15] and a subsequent increase in infectivity levels to near pretreatment levels 12 months later [8]. Hence, high treatment coverage and sustained compliance are given due emphasis in disease control programs of onchocerciasis in order to ensure its effectiveness.

Though compliance plays an important role in the success of onchocerciasis control, except for some anecdotes like perceived benefits and dangers of ivermectin treatment that are frequently cited as reasons for compliance and non-compliance respectively, there are no scientifically documented explanations that can provide a direct insight into why individual community members do or do not take ivermectin in the study area. In addition, only few similar studies could be found elsewhere [16-18] that can help answer the questions associated with compliance with CDTI.

This study, therefore, identifies factors associated with CDTI compliance and provides a basis for understanding how to sustain community control efforts over a long period to achieve success in the control of onchocerciasis as a public health and socioeconomic problem. This study attempted to answer one primary research question: (1) what factors are associated with CDTI compliance and what are the motivators and barriers of compliance with CDTI?

## Methods

### Setting

The study was conducted in Bebeka coffee plantation (farm), between February 1 and February 28, 2008. The study area is found Southwest Ethiopia, which is located 595 Kilometers South West of Addis Ababa. The area has a range of altitude between 900 and 1000 meters above sea level with annual rainfall of about 1728 millimeters. There are two major rivers in the area that are responsible for vector Simulidae (black flies) breeding namely; Aware and Gatcheb. The total population of the farm is 17164 (Bench-Maji zonal Economy & Finance Development Department, projected for the year 2007) and they reside in 20 villages.

As part of the control program, annual mass treatment has been started in the plantation since 2003 by World Health Organization (WHO)/APOC in partnership with Ethiopian Federal Ministry of Health, The Carter Centre, the local administration and the communities. Up to 2008 February, five treatment rounds have already taken place with CDTI strategy. Drug distribution is being carried out by CDDs under the supervision and technical assistance by health extension workers, nurses and health officers. Most of the villages have maintained complete and consistent annual treatment records using the APOC's treatment registration books. (Table 1)

**Table 1:** Population and Annual ivermectin Treatment Coverage of the Study Villages, Bebeka coffee plantation, Southwest Ethiopia, February 2008

Village name	Total no of population in the village	Individuals in the study	Annual ivermectin community treatment coverage				
			
			2003	2004	2005	2006	2007
Gatcheb	1179	94	66%	56%	51%	62%	75%
Awarie	350	47	70%	34%	64%	81%	87%
Hamsa-sidist	1113	79	63%	58%	72%	61%	68%
Nib-erbata	688	91	69%	62%	85%	77%	72%
Olme Gojeb	1684	145	52%	57%	61%	56%	63%

### Design

The study employed an unmatched case - control study design. Cases were, compliant i.e., those individuals who had been registered on the relevant treatment registers and had taken all the five annual doses of Ivermectin. Controls were, non-compliant, those individuals who had been recorded in the relevant treatment registers during the first treatment round (2003), and didn't take at least two doses of which one being in the last treatment round (2007). All individuals living in the study area and registered on the registration book since the first treatment round (i.e., 2003) are considered as source population for this study. Individuals exempted from treatment because of treatment ineligibility (i.e., under 5 years old or 90 cm tall; having serious health problem, e.g. asthma, pregnancy and lactation of infant less than one week of age) were excluded from the study. Those who are aged less than 15 years were excluded.

### Sample size and sampling procedure

During initial assessment, it was found that most of the villages (14 out of 20) have maintained complete and consistent annual treatment records using the APOC's treatment registration books. Five villages were selected randomly among the 14 villages included in the study. In the selected villages, there were 5014 individuals of which 2287 were found to be registered since 2003 for annual mass treatment. From the treatment registration books of the selected villages, all individuals who were eligible (1351) for the study were stratified into controls and cases. Meanwhile, cases and controls were listed separately on two different sampling frames to the size of 477 cases and 874 controls. After excluding those individuals who permanently left the area or died after the last treatment round from the list, a table of computer-generated random numbers was used to select the final cases (n = 228) and controls (n = 228) using simple random sampling.

The sample size was calculated using Epi-Info version 3.4.1 statistical software (Statcalc) by considering the following parameters: 95%CI, 80% power and 1; 1 ratio for controls and cases. By taking a significant risk factor from a Uganda study [16] i.e., 'perceived risk of having onchocerciasis with magnitude of 76% & 91% in non-compliers & compliers, respectively, the sample size required were 108 cases and108 controls. We also assumed a design effect of two and by adding 10% for non-responses in both groups; the total sample size was 456 (228 cases and 228 controls).

The selected cases and controls were then traced back in the community using their address and with the help of village leaders and CDDs. If the prospective interviewee was not present during the visit and could not be found even after three visits, he or she was considered a non- responder.

In-depth interviews using a semi-structured interview guide were conducted with eight key informants that were selected based on their affiliation with CDTI and the community, from health workers, community representatives and CDDs. The interview was conducted by a person who was trained and who had sufficient information about the program and the area.

### Measurements

The structured questionnaire, which was developed after an intensive literature review on the subject, was translated into Amharic and pre-tested on areas not included in the study. Compliance to ivermectin is defined as the extent to which individual members of the communities take ivermectin at each annual distribution since the inception of CDTI in the village. The definition is based on the number of dose(s) missed in specified treatment rounds, as reviewed from the treatment registration books. An individual was identified as compliant if he or she was registered on the registration book since the first treatment round (i.e., 2003), and took all five doses. On the other hand, an individual was identified as non-compliant if he or she was registered on the registration book since the first treatment round and had missed at least two of the five doses of ivermectin of which one non-treatment being in the last treatment round.

Data were collected by a pre-tested questionnaire, which was adopted, from different studies. A wide range of independent variables was utilized. Socio-demographic characteristics included: age categorized into two groups (≤35, and >35), sex, average monthly family income, education divided into three categories (no formal education, primary or less school, secondary and above), religion, marital status divided into two categories (married and currently not in marriage), employment status, duration of stay in the plantation (<10 years and≥10 years). Knowledge about onchocerciasis was measured with 11 questions emphasizing cause, transmission and prevention which assessed the knowledge of the individuals. Knowledge was scored by giving 0 for incorrect answers and 1 for correct answers. Perceived risk of onchocerciasis was measured with 3 questions. Each of the questions required a response on a four point scale (very high to very low). Perceived benefits of ivermectin are defined as the respondents' perception regarding the benefits of ivermectin. Respondents were said to have good/right perception if they mentioned that "ivermectin prevents onchocerciasis with or without mentioning expelling intestinal worms." Other responses were judged as wrong perception on the benefit of the drug. Attitude towards ivermectin was measured with 8 questions. An example of one such question is "ivermectin will eliminate the parasite causing onchocerciasis from the body". Response categories ranged from 1 to 4, for strongly disagree to strongly agree. Participation in community meetings to select CDD, perceived performance of community drug distributors and best method of dose determination were measured each by one question.

Potential factors associated with compliance were grouped into the socio-demographic characteristics such as age, gender, level of education and occupation, participant's behavioral factors such as knowledge, belief and attitudes towards the treatment, the disease, and the service, service related factors and social factors.

A semi-structured open-ended interview guide was used for the key informant interviews. The guide included questions which assessed the CDTI program, knowledge and beliefs related to CDTI compliance. On average each interview took one hour.

### Data Analysis

Data were coded and encoded using SPSS version 12.0.1 software. Also, data were cleaned and checked for outliers, inconsistencies and missing values. Knowledge was scored by giving zero (0) to incorrect answers and 1 for correct answers. Binary logistic analysis was used to compare socio-demographic characteristics. Odds ratio and 95% confidence interval (CI) were used to measure the strength of association between the potential factors and the outcome, i.e. compliance with CDTI. Variables that were significant at a level of P < 0.10 were considered for inclusion in multivariate models. Covariates were retained if they were significant at the 5% level. Finally, stepwise logistic regression with both forward likelihood methods were used to control possible confounders and to identify independent factors associated with CDTI compliance. The findings were consistent in both forward and backward likelihood methods.

All tape-recorded interviews were transcribed verbatim. The transcribed text from each informant was translated from Amharic to English. The data were transcribed and analyzed manually, in line with the objectives of the study. The translated text document of the note and the transcribed information were coded. Reading and coding were initiated while the data were being collected. The texts were read repeatedly to identify major themes. Finally an overall interpretation was done, about how thematic areas related to one another, explaining how the various concepts relate one another. These data were compared with the quantitative data, and some quotes from the qualitative data were integrated and presented in parallel with the quantitative information to elaborate the insights of the participants.

### Ethical Considerations

Ethical clearance was obtained from Jimma University ethical clearance committee of the faculty of public health. Permission was sought from Bebeka coffee plantation administration and the plantation's labor union. An informed verbal consent was obtained from every eligible individual before inclusion into the study by explaining the objective of the research. Privacy and confidentiality were ensured.

After the interview, data collectors provided important information regarding onchocerciasis mainly focusing on misperceptions and knowledge deficits observed during the interview with the respondents.

## Results

From the total of 456 individuals selected for administration of the survey questionnaire, 450 were contacted and completed the study. While two individuals refused to participate in the study, the remaining four were not available at home after repeated attempts, yielding a response rate of 98.7%.

Eight key informants were interviewed, two health workers, two community representatives and four community drug distributors (of whom two had dual responsibility as CDD and community leader). All were male, age above twenty five and except one all have lived in the area for more than twenty years. Only one health worker, age twenty six, has reported to have been living in the area for six years.

### Socio-demographic characteristics

Out of the 450 study participants, i.e., 225 cases and 225 controls, the majority (62.7%) of the cases and 72.9% of the controls were under 35 years of age and 56.9%of the cases and 52.0% of the controls were male in sex category as shown in Table 2. Both study groups i.e., cases and controls, were found to be similar in all but their age and employment status in that most of the controls (72.9%) were under 35 years of age showing a statistically significant difference from that of the cases (p = 0.03) and significantly higher proportion of cases (71.1%) were employed when compared to the controls (p = 0.01).

**Table 2:** Socio-demographic characteristics of cases and controls in Bebeka Coffee Plantation, Southwest Ethiopia, February 2008

Variable	Case Number (%)	Control Number (%)	p-value
**Age in years (Mean, SD, 31.4, 11.6)**	(32.7, 11.6)	(30.1, 12.1)	0.03*
≤35	141 (62.7)	164 (72.9)	
>35	84 (37.3)	61 (27.1)	
**Sex**			0.35
Male	128(56.9)	117 (52.0)	
Female	97 (43.1)	108 (48.0)	
**Current marital status**			0.27
Married	154 (68.4)	143(63.6)	
Currently not in marriage	71 (31.6)	82(36.4)	
**Highest grade completed**			
No formal education	66 (29.3)	67 (29.8)	0.77
Primary education	91 (40.4)	84 (37.3)	0.47
Secondary and post secondary	68 (30.2)	74 (32.9)	
**Employment status**			0.01*
Not-employed	65 (28.9)	90 (40.0)	
Employed	160(71.1)	135 (60.0)	
**Religion**			0.46
Christians	211 (93.8)	207 (92.0)	
Muslims	14 (6.2)	18 (8.0)	
**Ethnicity**			0.55
Endogenous ethnic groups	40 (17.8)	45(20.0)	
Non endogenous ethnic groups	185 (82.2)	180 (80.0)	
**Duration of stay in Bebeka (Mean, SD, 20.4, 6.5)**	(20.6, 6.8)	(20.2, 6.2)	0.63
< 10 years	16 (7.1)	14 (6.2)	
≥10 years	209 (92.9)	211 (93.8)	
**Monthly average family income(Ethiopian Birr)**			0.80
500 and less	186 (82.7)	188 (83.6)	
501 and above	39 (17.3)	37 (16.4)	

*Statistically significant at p-value <0.05, SD = Standard Deviation, 1$ = 9.7 birr

### Bivariate analysis

As depicted in Table 3 the bivariate analysis of this study revealed nine variables to be associated with compliance to community directed ivermectin treatment. Among the socio-demographic facts, respondents' age and their employment status showed significant association with the dependent variable.

**Table 3:** Variables associated with compliance in bivariate analysis, in Bebeka coffee plantation, Southwest Ethiopia, February 2008

Variable	Case No (%)	Control No (%)	COR (95% CI)	p-value
**Employment status**				
Not-employed	65 (28.9)	90 (40.0)	1.0	0.01
Employed	160(71.1)	135 (60.0)	1.64 (1.11-2.43)	
**Age**				
35 and lower	141 (62.7)	164 (72.9)	1.0	0.03
36 and above	84 (37.3)	61 (27.1)	1.60(1.07-2.39)	
**Perceived risk of having the disease**				
High	124 (55.1)	90 (40.0)	1.84 (1.27-2.68)	<0.001
Low	101 (44.9)	135 (60.0)	1.0	
**Family supports your intake of ivermectin**				
Yes	154 (68.4)	124 (55.1)	1.77 (1.20-2.60)	<0.001
Not accounted	71 (31.6)	101 (44.9)	1.0	
**Attitude towards the drug**				
Favorable attitude	140 (64.2)	100 (44.4)	2.06 (1.41-3.00)	<0.001
Unfavorable attitude	85 (37.8)	125 (55.6)	1.0	
**Knowledge about community directed treatment with ivermectin**				
Knowledgeable	88(39.1)	68 (30.2)	1.48 (1.01-2.19)	0.04
Not knowledgeable	137 (60.8)	157 (69.8)	1.0	
**Participated in community meetings to select community drug distributors**				
Yes	73 (32.4)	51 (22.7)	1.63 (1.07-2.48)	0.02
No	152(67.6)	174 (77.3)	1.0	
**Perceived performance of community drug distributors as**				
Well and very well	209 (92.9)	178 (79.1)	3.45 (1.89-6.23)	<0.001
Poor and very poor	16(7.1)	47 (20.9)	1.0	
**Measuring height is the best way of dose determination**				
Yes	221 (98.2)	194 (87.4)	7.97 (2.75-23.14)	<0.001
No	4 (1.8)	28 (12.6)	1.0	

COR = Crude Odds Ration, CI = Confidence Interval

### Multivariate analysis

A fully adjusted logistic regression model was developed that included nine exposure variables. As a result five independent predictors of compliance to CDTI were identified; employment, high risk-perception, family support, perceived performance of CDDs as good and believing the best method of one's dose determination is measuring height. As to the employment status, employed individuals were more likely to comply with ivermectin treatment when compared to unemployed individuals [AOR = 1.68, 05% CI 1.11-2.61] and those individuals who assumed themselves at high risk of getting onchocerciasis were almost twice as likely to comply with the treatment than individuals who have low or no risk perception [OR = 1.98, 95% CI 1.32-2.95] as shown in Table 4.

**Table 4:** Independent predictors of compliance with Community Directed Treatment with Ivermectin, Bebeka coffee plantation, Southwest Ethiopia, 2008

Variables	Case No (%)	Control No (%)	COR (95% CI)	AOR* (95% CI)	P-value
**Employment status**					<0.01
Not-employed	65 (28.9)	90 (40.0)	1.0	1.0	
Employed	160(71.1)	135 (60.0)	1.64 (1.11-2.43)	1.68 (1.11-2.61)	
**Perceived risk of having the disease**					<0.001
High	124 (55.1)	90 (40.0)	1.84 (1.27-2.68)	1.98 (1.32-2.95)	
Low	101 (44.9)	135 (60.0)	1.0	1.0	
**Family supports your intake of ivermectin**					
Yes	154 (68.4)	124 (55.1)	1.77 (1.20-2.60)	1.86 (1.22-2.84)	<0.001
Not accounted	71 (31.6)	101 (44.9)	1.0	1.0	
**Perceived performance of community drug distributors**					<0.001
Well and very well	209 (92.9)	178 (79.1)	3.45 (1.89-6.23)	2.84 (1.50-5.37)	
Poor and very poor	16(7.1)	47 (20.9)	1.0	1.0	
**Measuring height is the best way of dose determination**					<0.001
Yes	221 (98.2)	194 (87.4)	7.97 (2.75-23.14)	6.37 (2.10-19.29)	
No	4 (1.8)	28 (12.6)	1.0	1.0	

COR = Crud Odds Ratio, AOR = Adjusted Odds Ratio, CI = Confidence interval. * Adjusted for variables indicated in table 2.

Family support was another independent factor associated with compliance in this study with individuals perceiving that their families are supportive to take the treatment were 1.86 times more likely to comply with the treatment than those who do not perceive their families as supportive[AOR = 1.86, 95% CI 1.22-2.84].

Likewise, perceiving that the CDDs were doing their work well or very well was also identified as an independent factor associated with compliance. Individuals who perceived CDDs as doing their work well or very well are 2.84 times more likely to comply with the treatment than individuals who perceived CDD's performance as poor and very poor [AOR = 2.84, 95% CI 1.50-5.37]. Similarly the qualitative result of our study indicated poor performance of CDDs. A 45-year old community leader said "... the ability of drug distributors is under question mark. First, I don't think they have enough training on the disease and the treatments they are giving. They do not have better information than the community itself. This has been observed during the distribution times. The community members believed that they (CDDs) have sufficient knowledge and they usually ask different questions, and drug distributors sometimes give wrong or irrelevant answers. As a result, either they (the community members) will be misled or may lose confidence on the distributor (CDD)".

Finally, the strongest variable associated with compliance in this study was found to be the method of ivermectin dose determination. As shown in Table 3, individuals who perceived that measuring height is the best way of one's dose determination were 6.37 times more likely to comply with the treatment than individuals who perceived that measuring height is not the best way of one's dose determination [AOR = 6.37, 95% CI 2.10-19.29]. In our qualitative result most of the informants indicated that they were usually asked by community members about the correctness of measuring height to determine one's dosage.

A CDD, while explaining the issue said that:

".. I remember, individuals (members of the village) usually ask the correctness of measuring height for dose determination. We (drug distributors) always tell them that the method is approved and recommended by the health service. But there are some incidents in which I myself was puzzled. By measuring height, we sometimes give equal dose of the drug for people who are widely different in age and weight. I remember, for example, a mother that asked why she and her daughter are given equal number of tablets. Of course though they were equal in height, the mother was too fat and apparently older in age when compared to her very young and slender daughter. Both have taken three tablets. I don't think it (the drug) will work for the mother. In my opinion, it is not logical that people with different weight and age should take equal doses. The dosage might be different if weight was considered instead of height. I'm not still convinced about the accuracy of measuring height for dose determination."

With regard to the CDTI program, the experiences of the key informants concerning the communities' participation in community meetings that were (if any) intended to collectively select CDDs and determine date of the drug distribution. Accordingly, most of the key informants said that there were no special meetings in the communities on these issues but selection of the most of the CDDs was performed by the health service. One community representative, when explaining his experience about the selection process, he said that;

"... *individuals who were working in polio vaccination campaign were assigned by the health center workers to distribute the drugs; I don't remember special community meeting that was conducted to select these people"*.

Another key informant (health worker), said that;

"...*some communities have conducted meetings to select community drug distributors, but in some communities especially replacements of new CDDs by those who stopped up working as CDD was carried out by the health center and the near by health stations based on their willingness, ability to read and write and prior participation in other health activities."*

Regarding the date of drug distribution, all of the key informants similarly said that the distribution time was not decided by the community. The annual distribution activity takes place in almost similar times of the years, April or May, as the drugs are sent to the area from Zonal health department.

The key informants think that CDDs are performing well. However, the performance of CDDs is not with out question, the forty five years old community representative, when explaining about the CDDs performance said that;

"... *the ability of drug distributors is under question mark. First, I don't think that they have got enough training on the disease and the treatments they are giving. They do not have better information than the community itself. This has been observed during the distribution times. As the community members believe that they (CDDs) have sufficient knowledge, they usually ask different questions and drug distributors sometimes give wrong or irrelevant answers. As a result either they will be misled or may lose confidence on the distributor (CDD)"*.

On the number of days the distribution runs at each treatment round, the participants, especially the CDDs and the health workers, responded with wide variation. But as most of the key informants report, the distribution of ivermectin in the area runs for about a two week period. However health facilities continue to provide the treatment for about a month.

On the other hand, the opinions of the key informants on the factors that help individuals to comply with the treatment and reasons for non compliance were asked. According to the opinions of most of the key informants, the drug's ability in expelling intestinal worms, the communities' good perception on the treatment were considered to be important factors that help individuals to continuously take the treatment in line with the recommendations of the control program. As to the reasons for non-compliance, being absent during the treatment time, fear of side effects and lack of awareness both to the disease and the treatment were commonly cited by the key informants as a possible reasons.

## Discussion

This study has tried to identify factors that are associated with compliance to annual doses of ivermectin in the community directed treatment program of the Bebeka coffee plantation. Though few similar studies had been conducted in other African Program for Onchocerciasis Control (APOC) member African countries, it is the first in Ethiopia.

In the adjusted regression model, five variables were shown to be independently associated with compliance. These five predictors can be classified in three categories as; individuals' factors, social factors and program-related factors.

Among the socio-demographic variables, employment status was positively associated with compliance. This can be explained by the extent of mobility unemployed individuals show in the area. Particular to the study setting, it is customary that many of the unemployed individuals move to areas where casual business opportunities are available, like a gold mining area, located 60 km south of the farm, which attracts a lot of people from different parts of the country. The other possible explanation for association between non-compliance and unemployment can be associated with the reaction of students (in this case unemployed) with the treatment acceptance. However one can not exclude spurious association due to significant baseline difference between case and controls on employment status. Interestingly our qualitative analysis also showed that many students refuse to take the treatment in fear of side effects that could avert them from going to school as the drug's side effect may last up to one week. Moreover, a shorter period of ivermectin distribution that lasts for about two weeks in most communities possibly increases the likelihood of missing these highly mobile segments of the population, thus contributing to non-compliance.

The other factor positively associated with compliance in this study was high risk perception to the infection of onchocerciasis, which is consistent with another study [16]. It is a well-established fact that the likelihood of an action like treatment intake would increase if the perceived threat of the disease is high [19]. On the other hand, qualitative results of this study have shown that health education activities in the area were very weak that otherwise could provide epidemiological information that could have probably raised perceived risk of individuals to the disease.

Family members are indicated to be the primary component of social support network [20,21] where social support is an interpersonal process that involves exchange of information. Information as a means to an end consists of facts, advice, words of reassurance [22], positive affirmation [20-22], which affects individual behaviors and decisions including compliance to medication. Similarly support of family members to participate in treatment was also significantly associated with compliance in our study. This was consistent with other study conducted elsewhere in the region [16].

Our findings also showed that perceived good performance of CDDs by the population is associated with increased compliance. This result is similar to what other investigators have found out elsewhere [16,17]. It is a matter of fact that CDDs, to be able to carry out their tasks, need re-training [23]. This shows that their role as efficient drug distributor and community health educator is emphasized in the program. However, our qualitative study that included CDDs themselves revealed insufficient knowledge on the side of CDDs that might have resulted in hampered acceptability by the community. It was also clearly indicated that the community members had poor confidence and trust on CDDs having perceived their performance as incompetent. Some community members apparently cited lack of trust and confidence on the CDDs as reason for non compliance. Moreover, some of CDDs admitted low performances (de-motivation) related to the interruption of incentives that were provided at the beginning of the program. The incentives that included financial incentives, T-shirts and other necessary supplies are no longer supplied since the last two to three rounds as described by the CDDs.

The strongest factor associated with increased compliance in this study was found to be belief on the use of measuring height as a proper way to determine somebody's dose. This concurs with the results from Uganda [16]. The authors attributed to the "belief in this population (particularly among non-compliers) that tall people are normally weak and therefore risk being overdosed if height instead of weight is used as a criterion for determining one's dose of ivermectin".

Similarly our qualitative data showed the communities' beliefs in that using height for dose determination makes different people with wide variation in weight receive equal doses of ivermectin they are taking, which may result in incorrect dosage. This can be an indication of the level of health education in the study area. Therefore it is important to strengthen health education activities in the project areas to dispel myths such as this.

The results of this study should be interpreted cautiously. First, the study was conducted among a plantation setting, which is structurally different from the traditional communities. This indicates that the results cannot necessarily be generalized to other non-plantational communities. The selection of cases and controls entirely depend on the CDTI register book which may result in misclassification bias. There were significant differences between cases and controls with respect to age and employment status. Although adjustment for these factors did not appreciably affect the association, we cannot rule out residual confounding. Finally we have not measured history of onchocerciasis which might influence compliance. With the aforementioned limitations in mind, we conclude that our findings have implications for interventions with onchocerciasis control program in the area and other similar setups.

## Conclusion

As programs to control onchocerciasis become more developed and have completed several annual ivermectin treatment rounds, it may become necessary to address the issue of non-compliance through innovative public health approaches. Designing interventions that reduce the number of non-compliant persons might make the difference between successful control of transmission of onchocerciasis, and mere control of the parasite. To that end; the distribution should last long enough, at each treatment round, in order to reach less compliers (eg. more mobile unemployed village members.), health education should focus on epidemiological information in order to increase risk perception and should target family members. In addition health education should also incorporate information regarding the use of measuring height as it is equally reliable with measuring weight in determination of ivermectin dose that somebody should take. Finally motivation and continual support to improve CDD's performance including training and incentives are crucial.

## Competing interests

The authors declare that they have no competing interests.

## Authors' contributions

DY conceived the study and analyzed and wrote the paper KD, KW, WK and MW involved in designing the survey and undertook preliminary analysis and involved in writing the paper. All contributed to the final report.
